# Characterization of female reproductive disturbances post-traumatic injury in *Drosophila melanogaster*

**DOI:** 10.17912/micropub.biology.000883

**Published:** 2023-09-19

**Authors:** Cameron Dixon, Kimberly McCall

**Affiliations:** 1 Molecular Biology, Cell Biology, and Biochemistry Graduate Program, Boston University, Boston, Massachusetts, United States; 2 Department of Biology, Boston University, Boston, Massachusetts, United States

## Abstract

Traumatic injuries (TIs) from intimate partner violence, vehicular collisions, high-impact sports, and even mundane activities can be fatal. However, survivors of TIs can have pathophysiological disturbances post-injury, including neurodegenerative diseases, mental illness, and metabolic disorders.Reproductive issues are a known consequence of TI especially in women, however this has remained poorly understood. *Drosophila* *melanogaster* has recently emerged as a stellar model of TI, however reproductive consequences have not been reported. Using the *Drosophila* model, we find reproductive consequences in the form of decreased egg laying and the retention of mature egg chambers mimicking issues in ovulation. These findings indicate that reproductive consequences of TI are conserved between *Drosophila* and humans.

**Figure 1. Drosophila can serve as a model for reproductive defects post-trauma:**
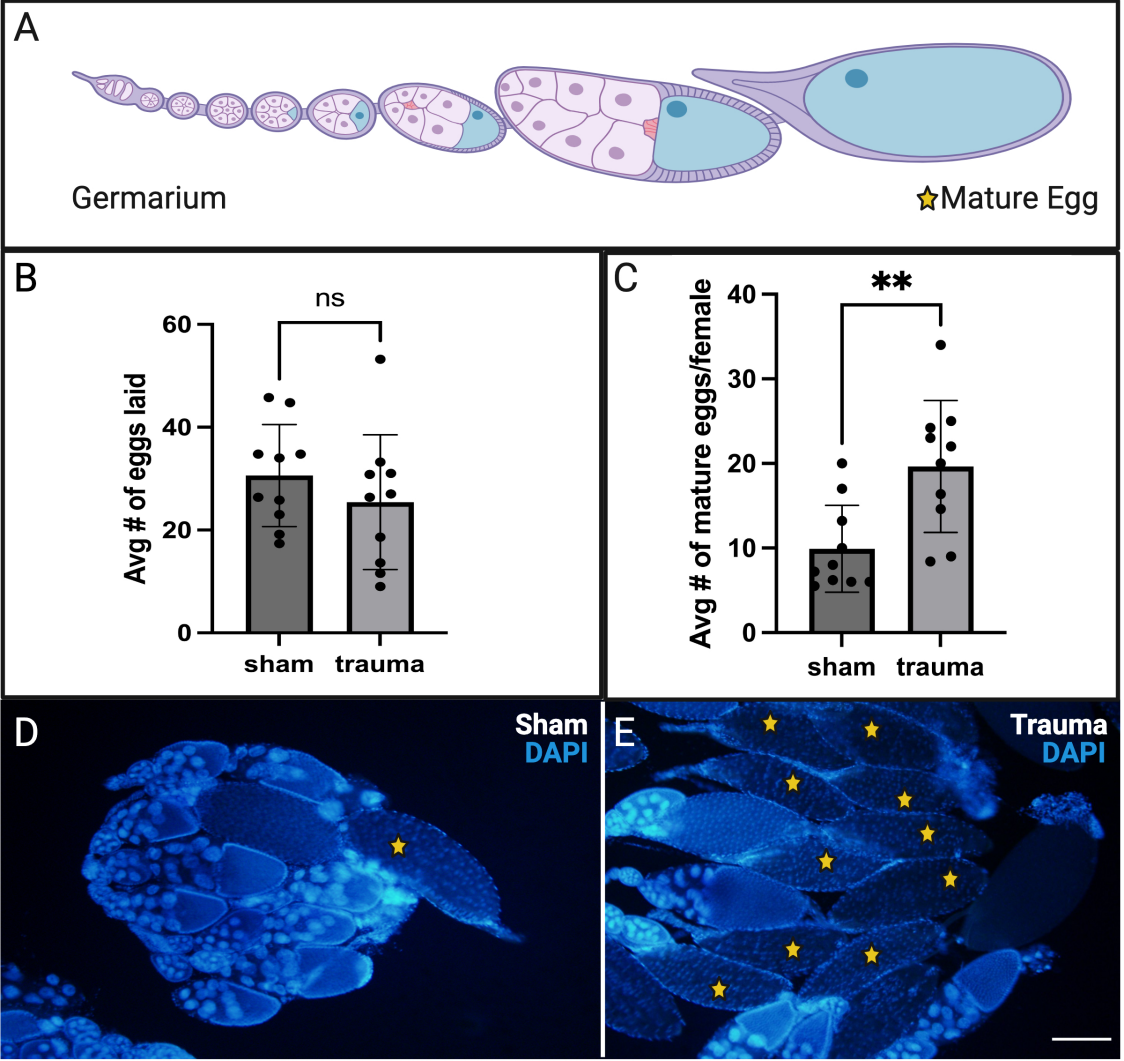
A. Schematic depicting an ovariole of the *Drosophila* ovary showing a string of developing egg chambers originating from the germarium (stem cell niche). B. Quantification of number of eggs laid per female per day (over a 5d period) showed a slight decrease (not significant via unpaired student t-test). C. Quantification of mature eggs shows an increase of retention in trauma females (** significant via unpaired student t-test, p=.0040). D. Ovaries dissected from sham female (6 days post trauma (dpt)). E. Ovaries dissected from trauma females (6dpt). Mature eggs in D and E are marked by stars. Scale bar represents 200 µm.

## Description

Physiological consequences post-traumatic injury (TI) including neurodegeneration, metabolic disorders, and reproductive issues have been observed in animal models, mimicking the effects in humans (Bansal et al. 2009, Ripley et al. 2008, Shi et al. 2016) . For vertebrates, mice and zebrafish have been utilized to investigate the complex organismal responses to TI. Reproductive issues following TI have been reported in humans, specifically females, and these issues arise through menstrual dysregulation, decreases in fertility and libido, and increased rates of miscarriages, however the links between TI and reproduction are not fully understood (Ripley et al. 2008) . In mice, brain specific TI instigates improper activation of the hypothalamic-pituitary-adrenal (HPA) axis, more specifically in females, which is thought to play a role in sex differences in response to TI (Tapp et al. 2019) .The dysregulation of the HPA axis leads to improper control over hormonal signaling. This breakdown in regulation may explain issues with reproduction, however many gaps remain in understanding the reproductive consequences of TI.

*Drosophila* *melanogaster* has emerged as a powerful model of TI due to relatively short lifespan, conservation of molecular/cellular cascades following injury response, and avoidance of the ethical dilemmas of inducing TI in mammalian models. However, reproductive consequences of TI have not previously been reported in the *Drosophila* model. The female reproductive system of *Drosophila* consists of two ovaries which each contain approximately sixteen ovarioles. Ovarioles are assembly lines of fourteen stages of developing egg chambers beginning at the germarium and ending at the mature egg that is ready for fertilization ( Figure 1A ) (Spradling 1993) . Given the evolutionarily conserved impacts of TI, we investigated whether *Drosophila* had reproductive consequences after TI.

*Drosophila* exhibit many different types of trackable reproductive behaviors. Egg laying is a behavioral assay that is affected by oocyte development and decision-making behavior that involves female flies choosing a viable environment for their progeny (Knapp et al. 2017) . To test whether this hallmark reproductive behavior is affected by TI, flies were given TI using a High Impact Trauma (HIT) device (Katzenberger et al. 2015) , and following a brief recovery period, egg laying was measured in TI and sham flies over a five-day timecourse. We found a consistent decrease in the number of eggs laid following TI, although it was not statistically significant ( Figure 1B ).

To further investigate impacts on reproduction, ovary morphology was examined. Ovaries were examined after the egg laying assay had concluded and the same females were taken for dissections and microscopy. Visualization of ovary morphology showed a significant increase in the retention of mature eggs ( Figure 1C -E), an indication that ovulation is disrupted (Nässel et al. 2020).

With the identification of reproductive effects in *Drosophila* following TI, questions arise such as 1) Will aging flies that have undergone trauma exhibit increased retention or develop other reproductive phenotypes? 2) What signaling mechanisms trigger these reproductive issues post-trauma? 3) What are the effects of diet on reproduction post-trauma? Age-related effects are of particular interest since consequences of trauma are known to develop both short and long term from the timepoint of injury (Shi et al. 2016) . While we found an onset of reproductive consequences about 72 hours post trauma, this is only a small window and not a clear overview of the severity of reproductive consequences. An aging timecourse experiment would help elucidate the progression of the ovulation defect that we have already found but also detect the onset of any other phenotypes that may have not had enough time to develop in the original five-day timecourse.

Investigation into possible genetic and cellular mechanisms behind these ovulation defects could provide insight into similar effects in humans. For example, a hormonal link is an intriguing candidate for this change in egg laying and retention. Hormonal signaling and function is highly conserved in *Drosophila* and it has been shown that alteration can affect ovary health (Knapp et al. 2017, Nässel et al. 2020). Identification of hormonal differences post-trauma could explain these reproductive issues found in females.

## Methods

Fly Strains

Flies were raised on a cornmeal-yeast diet at 25°C with 12 hour day-light cycle. The *w ^1118^* strain, which contains a loss of function allele in the *white* gene and is a commonly used genetic background strain, was used for all experiments. These flies were separated into groups based on whether they received trauma or were sham controls. All groups were conditioned with wet yeast paste two days prior to the date of trauma and were supplied fresh yeast paste daily.

High Impact Trauma (HIT)

The HIT device was constructed in house following published guidelines. Usage of device was in congruence with Katzenberger R et al (2015). All experiments were done at 90° and trauma was applied once to mimic mild TI.

Egg Laying Assay

Male and female flies were raised together at 25C° and aged 5 days post eclosion prior to trauma. Groups of females were then collected and subjected to the HIT device or treated as a sham group. Females were then reunited with the males that they were originally paired with. After recovery (24 hours post trauma), groups of 5 females with 5 males were placed into an egg laying apparatus consisting of an inverted fly bottle over a grape juice agar plate supplemented with a small dab of yeast paste. Plates were replaced daily and eggs were counted using a dissecting microscope. This assay was done in replicate ten times. Grape juice agar media was made by mixing 30g of Agar (Difco) in 1L of H _2_ O and autoclaved. Once cooled to 65°C, 40g of sucrose dissolved in 400 mL of Grape Juice and 20ml of 10% Tegosept (in EtOH) were added.

Dissection/Microscopy

Ovaries were dissected in 1x phosphate buffered saline with 0.1% Triton-X (PBT). Tissue was then fixed for 20 minutes in 600uL of PBT and 200uL of 16% EM grade paraformaldehyde. Samples were washed with PBT three times and incubated in Vectashield with DAPI (Vector Labs). Stage 14 mature egg chambers (identified by presence of dorsal appendages) were visualized and scored on an Olympus BX60 microscope. Mature eggs were quantified and compared between sham and trauma groups. Each experiment was performed ten times consisting of five females each. Statistical analysis was performed using GraphPad Prism (unpaired student t-tests). Images were processed in FIJI and the figure was created using BioRender.com.
